# Seawater‐Degradable Polymers—Fighting the Marine Plastic Pollution

**DOI:** 10.1002/advs.202001121

**Published:** 2020-11-23

**Authors:** Ge‐Xia Wang, Dan Huang, Jun‐Hui Ji, Carolin Völker, Frederik R. Wurm

**Affiliations:** ^1^ National Engineering Research Center of Engineering Plastics Technical Institute of Physics and Chemistry The Chinese Academy of Sciences Beijing 100190 P. R. China; ^2^ University of Chinese Academy of Sciences Beijing 100049 P. R. China; ^3^ ISOE – Institute for Social‐Ecological Research Hamburger Allee 45 Frankfurt 60486 Germany; ^4^ Max‐Planck‐Institut für Polymerforschung Ackermannweg 10 Mainz 55128 Germany; ^5^ Sustainable Polymer Chemistry Group MESA+ Institute for Nanotechnology, Faculty of Science and Technology, Universiteit Twente PO Box 217 Enschede 7500 AE The Netherlands

**Keywords:** biodegradability, biodegradable polyesters, marine plastic pollution, seawater‐degradable polymers

## Abstract

Polymers shape human life but they also have been identified as pollutants in the oceans due to their long lifetime and low degradability. Recently, various researchers have studied the impact of (micro)plastics on marine life, biodiversity, and potential toxicity. Even if the consequences are still heavily discussed, prevention of unnecessary waste is desired. Especially, newly designed polymers that degrade in seawater are discussed as potential alternatives to commodity polymers in certain applications. Biodegradable polymers that degrade in vivo (used for biomedical applications) or during composting often exhibit too slow degradation rates in seawater. To date, no comprehensive summary for the degradation performance of polymers in seawater has been reported, nor are the studies for seawater‐degradation following uniform standards. This review summarizes concepts, mechanisms, and other factors affecting the degradation process in seawater of several biodegradable polymers or polymer blends. As most of such materials cannot degrade or degrade too slowly, strategies and innovative routes for the preparation of seawater‐degradable polymers with rapid degradation in natural environments are reviewed. It is believed that this selection will help to further understand and drive the development of seawater‐degradable polymers.

## Graphical abstract

Plastic pollution of the oceans is a major concern today due to the long life of commodity polymers. The degradation profiles of conventional biodegradable polymers, such as polylactide, polycaprolactone, and others in seawater, are reviewed. As many of them degrade relatively slowly, additional strategies for the development of seawater‐degradable polymers are highlighted.

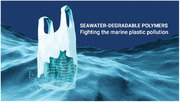

## 1 Introduction

We are living in a polymers age. Since Staudinger coined the term “macromolecule” in 1920,^[^ ^1^ ^]^ polymers have shaped our world in lightweight products, high‐performance materials, everywhere in our daily life. Initially intended to be a versatile material for the broad community (“Nylon instead of silk”), our society has produced excessive amounts of plastic products, many of them for packaging. This massive plastic production of more than 8.3 billion tons ever produced, has led to worldwide plastic pollution.^[^ ^2^, ^3^ ^]^ From this amount, 4.8–12.7 million tons of plastic waste are directly discarded or delivered through rivers or by the wind into the oceans every year.^[^ ^4^ ^]^ Today, various types of plastics have been identified in the ocean. A recent meta‐analysis confirmed that the most abundant plastic types polluting marine environments are polyethylene (PE, 23%), and polyesters/polyamide, (20%), followed by polypropylene (PP, 13%) and polystyrene (PS, 4%), accounted for 74% of global plastic production in 2015 (commonly used in short life‐cycle products).^[^ ^5^ ^]^ These polymers are hardly degradable in marine but can continually fragmented into small pieces by physical, chemical, or biological effects such as ultraviolet rays, weathering, ocean currents and form “microplastics,” which are heavily discussed today.^[^ ^6^, ^7^ ^]^ Due to the persistence of polymers, complete biodegradation of such marine plastic waste is expected to take decades or even centuries.^[^ ^8^ ^]^ Although the impact of marine plastic pollution on the complex marine ecosystem still needs further studies, effects on growth, development, ability to avoid natural enemies and reproduction of marine organisms have been proven in laboratory conditions.^[^ ^9^ ^]^ Larger animals such as turtles and seals can be suffocated by discarded plastic threads or nets. Birds, fish, mollusks, and other (marine) organisms take up small plastic fragments. Habitats such as coral reefs or mangrove forests are damaged. Due to its durability, plastic debris can travel long distances with the ocean currents and are therefore also found in the arctic regions. As a result, they may carry foreign species and potential pathogens to endanger the stability of marine ecosystems, and further enter the terrestrial food chain through air, drinking water, salt, seafood, and so forth, which might also influence human health.^[^ ^10^ ^]^

Marine plastic waste pollution has been listed as one of the top ten environmental problems to be solved globally since the first UN Environment Conference in 2014. In 2018, the United Nations Environment Program issued the theme of World Environment Day, “Beat Plastic Pollution,” calling on all countries in the world to work together to fight the problem of plastic pollution. The 2018 European Commission issued the “Plastic Strategy in the European Circular Economy,” which proposes that more than half of the plastic waste in the European market to be recycled by 2030. Today, more than 15 countries/regions around the world have successively issued policies to “ban plastic” and “limit plastic” including India, New York State and the Washington State of American, EU member states, Hainan and Jilin provinces of China. The existing measures focus on reducing, reusing and recycling plastics, which aim stopping the plastic‐waste problem on land before plastic waste is washed into the oceans, however, this highly relies on the enforcement of government and a raised environmental awareness of public.^[^ ^11^ ^]^ Although the scientific and the public awareness on the problem of plastic waste increased in many countries, numerous actions tackling plastic accumulation by encouraging active involvement of consumers, producers, industry, and companies are discussing in the media, active measures still need to be implemented for a sustainable future of polymer packaging. Today various attempts can be found in the literature and social media, on local measures to clean up seawater, either with machines or by the hands of volunteers.^[^ ^12^ ^]^ However, these strategies cannot remove marine microplastics in the vast of the oceans, not only due to their small size but obviously due to the widespread and large volumes which would be needed to be cleaned up.^[^ ^2^, ^7^ ^]^

In addition to sustainable consumption and use of plastics as well as improved recycling or waste management, plastic degradation technology may be a promising option, and which in 2019 has been identified by IUPAC as one of the 10 chemical innovations that are most likely to change human society in the future. This technology involves two aspects of research. On the one hand, the development of efficient plastic degradation technology, especially some novel biotechnological approaches for the sustainable biological degradation of mixtures of both recalcitrant and degradable plastics.^[^ ^13^ ^]^ This also includes the development of environmentally friendly and sustainable solutions for managing the waste of plastics mixtures based on the use of communities of microorganisms with a set of complementary enzymes.^[^ ^14^ ^]^ On the other hand, search and application of seawater‐degradable alternatives to normal non‐degradable polymers might be another strategy to prevent any accumulation of plastic if littered and ended up in the ocean, which will be summarized in this review.

To date, many types of biodegradable polymers have been synthesized in the laboratory, but from the perspective of industrialization and cost considerations, processing, and mechanical properties of current production and application, there are only few commodity products. The majority of them are aliphatic polyesters, which can undergo hydrolysis in water or by microorganisms. Enzymatic degradation of these polyesters can occur in the compost or the soil over periods of several months, resulting in full biomineralization.^[^ ^15^, ^16^, ^17^ ^]^ At the first glance, biodegradable materials might be the way to solve the marine plastic pollution. However, many of them do not degrade in seawater or only with very slow degradation rates.^[^ ^18^, ^19^, ^20^, ^21^, ^22^ ^]^ In general, the degradation process is affected by the intrinsic factors of the polymer, such as the chemical structure, crystallinity, molecular weight, shape, and size of the products.^[^ ^23^, ^24^, ^25^ ^]^ Moreover, external environmental factors, such as types and amount of microorganisms, temperature, UV exposure, pH, and salinity in different waters can also influence the rate of biodegradation.^[^ ^26^, ^27^, ^28^ ^]^ To date, no comprehensive evaluation of the biodegradation of certain polymers in seawater has been presented. It is thus essential to re‐examine the degradation behavior of polymers in the marine environment and establish standards, in order to facilitate the decision if and which biodegradable polymers should be included in bans and taxes on plastic, and also guide research and development to design and develop safe and reliable seawater‐degradable materials.

**Figure 1:**
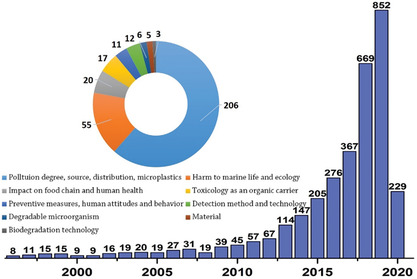
The number of publications about marine plastic pollution (data collected from Web of Science, March 20, 2020).

Using “marine plastic pollution” as the keyword to search in Web of Science, 3449 related documents appeared (Mar. 20/2020). With the first scientific paper on plastic pollution in the ocean by Carpenter and coworkers in 1972,^[^ ^29^ ^]^ it took several decades until the plastic pollution became a focus of modern research. The number of documents in 2019 outreaches all publications of the previous years. By analyzing 336 review articles, we found that most of the research covers the source, volume, and impact of marine plastic pollution on life, with a special focus on microplastics (**Figure** **1**). Preventive measures mostly concern human attitudes and behavior.^[^ ^30^, ^31^ ^]^ To date, no review article summarized strategies for design and synthesis of seawater‐degradable polymers.

Besides, the end‐of‐life solution to marine plastic pollution is rare and only appeared in a few articles, in which biodegradable polyesters had been discussed.^[^ ^32^, ^33^, ^34^ ^]^ In a recent article, Haider et al. summarized data on the biodegradation of promising degradable polymers under natural conditions including marine waters, soil, and compost and highlighted that biodegradable polymers are often not as “biodegradable” as they claim to be.^[^ ^32^ ^]^ Especially polylactide (PLA), almost a commodity today, did not show obvious signs of degradation after 1 year in seawater.^[^ ^21^ ^]^

In this review, we have collected the published data on the degradation performance of the most common biodegradable polymers in seawater, that are PLA, poly(butylene adipate‐*co*‐terephthalate) (PBAT), polybutylene succinate (PBS), polyhydroxyalkanoate (PHAs), and poly(*ε*‐caprolactone) (PCL) and some others. We also summarized recent research examples for the construction of quickly seawater‐degradable polymers and their blends. We want to answer the following questions: Can existing biodegradable plastics replace commodity plastics to solve the problem of plastic pollution in the ocean? If not, what should we do next for a safe and available marine friendly material? It will be essential to investigate the degradability of various polymers in seawater and to assign prerequisites for biodegradation in seawater (in a reasonable timeframe) and to ascertain the possible applications for such seawater‐degradable polymers.

## 2 Biodegradable Polyesters

### 2.1 Overview: Property, Market, and Application of Biodegradable Polyesters

**Scheme 1:**
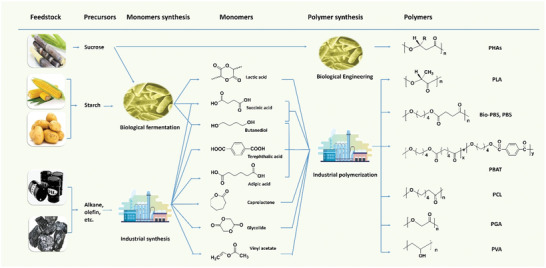
Structures and sources of commercial biodegradable polymers (bio‐based and fossil‐based).

Commercial biodegradable polymers can be divided into three categories according to their raw materials and synthetic methods (**Scheme** **1**).^[^ ^17^, ^35^ ^]^ The first category is biodegradable plastics obtained from renewable materials such as polyhydroxyalkanoates (PHAs), for example, poly(3‐hydroxybutyrate) (P3HB), poly‐3‐hydroxyvalerate (PHV), and their copolymers poly(3‐hydroxybutyrate‐*co*‐3‐hydroxy valerate) [P(3HB‐*co*‐3HV)], poly(3‐hydroxybutyrate‐*co*‐4‐hydroxybutyrate) [P(3HB‐*co*‐4HB)], which are produced by a microbial fermentation process. The second category is biodegradable plastics synthesized by industrial processes using renewable monomer precursors, including PLA, bio‐based PBS and poly(butylene succinate‐*co*‐butylene adipate) (PBSA). The third category is biodegradable plastics synthesized from petrochemical resources, including PBS, polycaprolactone (PCL), poly(butylene adipate terephthalate) (PBAT), polyglycolide (PGA), PBSA, poly(propylene carbonate) (PPC), poly(vinyl alcohol) (PVA), and others. At present, with the increasing awareness of global pollution, the demand for biodegradable plastics has grown, and the cost has gradually approached that of commodity plastics, which led to a replacement of some commodity plastics already. An annual production capacity of 1.17 million tons in 2019 of biodegradable polymers was reported.^[^ ^36^ ^]^

Among all biodegradable materials, the most four productive materials are starch, PLA, PBS, and PBAT, which accounted for 38.4%, 25.0%, 7.7%, and 24.1% of the total biodegradable plastic capacity (according to the European Bioplastics Data in 2019).^[^ ^36^ ^]^ Starch is a widely used natural degradable polymer material with unique biodegradability in different natural environments. It is usually plasticized and blended with other polymers to increase the bio‐based content of the product or to reduce costs.^[^ ^37^, ^38^ ^]^ With the highest production numbers of 449000 tons per year, PLA is used in many fields such as disposable tableware, medical applications, packaging, and so forth.^[^ ^36^ ^]^ The bright development prospect of PLA was not only because of its excellent mechanical strength and unique transparency but also because that the feedstock lactic acid is renewable and PLA is considered as the “green” material.^[^ ^39^, ^40^ ^]^ The inherent high *T*_g_ makes them brittle at room temperature and difficult to blow into thin films, this together with its poor thermal resistance has been the major bottleneck for its large‐scale commercial applications.^[^ ^41^ ^]^ The emergence of PBS and PBAT just makes up for this shortcoming. The biggest advantage of PBS is that it combines mechanical strength and toughness while being resistant to hot water. This makes it suitable for many applications, except those that have high requirements for barrier properties.^[^ ^42^ ^]^ Also, PBS can be considered a biomaterial because the feedstock succinic acid can be prepared from biomass such as corn or soybeans by biological fermentation.^[^ ^40^, ^43^ ^]^ The most important feature of PBAT is its excellent film‐forming properties while ensuring mechanical strength. It can be used to prepare a variety of disposable film products, including bags or is especially appealing for the use as agricultural mulch films.^[^ ^44^ ^]^ It is worth mentioning that due to the low cost of terephthalic acid, the production cost of PBAT is the lowest among biodegradable polyesters (≈1500 €/ton). With the increasing use of biodegradable materials in the field of disposable packaging, the production capacity of PBAT and its market share among all biodegradable plastics rise gradually from 11.6% in 2017 to 16.7% in 2018, then to 24.1% in 2019.

Other polyesters, such as PCL, a fossil‐ based semicrystalline polyester can undergo biodegradation by both aerobic and anaerobic microorganisms in most natural environments. However the relatively low *T*_m_ (58 °C), poor temperature resistance and high costs limit its application range.

In contrast, naturally produced PHAs are considered as a unique class of commercially implemented bio‐based biodegradable and/or biocompatible polyesters, which perform a wide range properties depending on the length of the side aliphatic chain at the *β*‐carbon. However, owing to the high production costs and complexity of extraction processes, the PHAs market and applications are still small but quickly growing with a market worth of 57 million US$ (2019), which is projected to be 98 million US$ in 2021.

### 2.2 Biodegradation: Description and Key Factors

**Figure 2:**
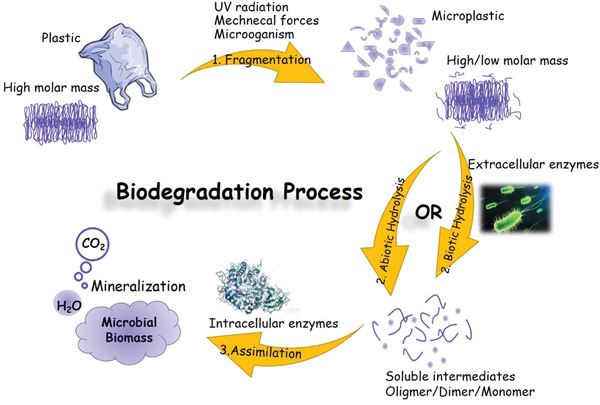
Schematic representation of the different steps involved in biodegradation.

“Biodegradable polymers and plastics are materials that by the action of microorganisms are quantitatively converted either to CO_2_ and H_2_O or to CH_4_ and H_2_O, respectively, under aerobic or anaerobic conditions.” This definition of biodegradable plastics was given by ASTM committee D‐20, the Committee on Plastics, formed a Subcommittee on Degradable Plastics, D‐20.96.^[^ ^17^ ^]^ Biodegradation is an enzymatic hydrolysis process catalyzed by microbial secretases, which is often divided into three stages:^[^ ^45^, ^46^ ^]^ fragmentation, hydrolysis and assimilation (**Figure** **2**). Firstly, polymers are fragmented into small pieces or microplastics by weathering, UV‐irradiation, mechanical forces, microorganisms, and so forth. Then, hydrolysis takes place at the ester bond of the polymer and eventually leading to the reducing molar mass and the formation of soluble oligomers, dimers, and monomers. Finally, these degradation products are taken up and used as carbon sources and energy by intracellular enzymes to produce increased cell biomass and simple end products like CO_2_ and water, named as bioassimilation and mineralization process. To reach the final process two prerequisites are essential: 1) the presence of certain polymer degrading microorganism; 2) the polymer undergoes hydrolysis to depolymerize into small enough fragments (such as oligomers, dimers, and monomers) to enter the microbial cells.

The second hydrolysis process is considered as the rate limiting step as the bioassimilation and mineralization process is assumed to be rapid.^[^ ^47^ ^]^ The hydrolysis of the polymer can either occur biotic or abiotic, in which the abiotic hydrolysis is 8–20 orders of magnitude slower than the enzymatic hydrolysis.^[^ ^46^, ^48^ ^]^ The rate of hydrolysis highly related to internal factors such as the chemical structure, molecular weight, the chain flexibility, crystallinity, regularity and heterogeneity, functional groups, and size of the material. Both high crystallinity and low hydrophilicity will hinder the penetration and attack of water, resulting in a slow hydrolysis process.^[^ ^49^ ^]^ Even with the same structure, molecular weight and size can severely affect the rate of hydrolysis.^[^ ^24^, ^25^ ^]^ Besides, the external environment, such as temperature, moisture, pH, UV, and the population of active microorganisms are also essential.^[^ ^27^, ^50^, ^51^ ^]^ Although many biodegradable polyesters are hard to hydrolyze and predict the lifetime when immersed in abiotic water, accelerated hydrolysis is often used to analyze degradation behavior of such polymers, either by accelerated hydrolysis, for example, by changing pH values or increasing the temperature, or by incubation the samples into an environment, which is rich in microorganisms, such as soil, activated sludge, or compost.^[^ ^50^, ^52^ ^]^

The biodegradation of polyesters is typically assessed by such accelerated conditions or certified for industrial composting. For example, PLA degrades slower (times vary drastically from report to report) in landfill or natural soil at ambient temperatures but can completely biodegrade within 6–9 month in industrial compost,^[^ ^28^, ^50^ ^]^ because the abiotic hydrolysis depends dramatically on the temperature and humidity.^[^ ^46^ ^]^ The temperature in industrial composts is higher (58–65 °C) and close to its *T*_g_, which increases the hydrolysis rates.

It is worth mentioning that microorganisms are specific to the degradation process of polyesters, certain enzymes can degrade specific bonds, and the specificity of the hydrolase substrate can greatly affect the observed rate of hydrolysis.^[^ ^53^, ^54^ ^]^ As a result, unlike abiotic hydrolytic degradation, enzymatic degradation is not common in synthetic polymers as a consequence of the substrate‐specific nature of many enzymes.^[^ ^55^, ^56^ ^]^ In addition, extracellular enzymes produced by different microorganisms may have active sites with different specificities, such as complementary shape, charge, and hydrophilicity/hydrophobicity, and hence have more capability to biodegrade certain polymers.^[^ ^15^ ^]^

## 3 Degradation of Polyesters in Seawater

Today, polyesters are probably the most promising class of material to undergo degradation in seawater. However, the term “biodegradable” often neglects the necessary for certain conditions, for example, the internal and external effecting factors of the material degradation process, which might result in a very different seawater degradation for many polyesters. The degradability of plastics in seawater is complex and requires an in‐depth investigation and evaluation to provide an accurate basis for the practical application of materials.

### 3.1 Environmental Characteristics of Seawater

Compared to soil and compost, the marine environment is characterized by low temperature, high salinity, high pressure, currents, and low nutrient levels (e.g., nitrate). In addition to temperature variation between different seasons and different areas, the water temperature varies with the depth in the vertical direction. The average surface temperature of seawater is 17.4 °C.^[^ ^57^ ^]^ The temperature drops to 0–4 °C when the water depth exceeds 2000 m.^[^ ^58^ ^]^ Seawater is rich in inorganic salts, and salinity is related to evaporation, precipitation, river runoff, and seawater currents. In different areas, the salinity varies at different depths, or at the same sea area shows seasonal changes. The salinity of the offshore and estuary waters generally does not exceed 30%, and the surface salinity of the ocean is between 32% and 37%, with an average of 35%. Seawater is weakly alkaline with pH values of ≈8.0 and 8.5. The surface seawater is usually stable at pH = 8.1 ± 0.2, while the middle and deep seawater generally varies between 7.8 and 7.5. The dissolved oxygen content in seawater is significantly lower than that in the soil, both of which are affected by temperature and closely linked to biological processes, for example, the presence and abundance of certain marine organisms. Similar to the abiotic factors (e.g., salinity, temperature, pressure, UV radiation), the biological habitat in the marine environment is vertically structured and shows different species compositions depending on the zones (e.g., the epipelagic zone (0–200 m), which is influenced by UV radiation and currents and the depth, or abyssopelagic zone, from around 4000 m down to the ground, which is completely dark, shows high pressure and low temperatures).

Microorganisms are an important group of diverse (mostly unicellular) organisms including bacteria, archaea, unicellular algae, fungi, and protozoans. They are ubiquitous in the various marine habitats and an important part of marine food webs. Autotrophic microorganisms (e.g., algae, cyanobacteria) produce organic matter in the upper, light‐flooded layers (epipelagic zone) by photosynthesis. Deeper zones without UV radiation are mostly inhabited by heterotrophic organisms that decompose organic substances (mostly heterotrophic bacteria and to a lesser extent fungi). They settled on dissolved organic matter or particulate organic matter (DOM or POM), but also on marine debris, that is, larger organic material including fragmented plastics. Marine ecosystems contain about half as many microorganisms as terrestrial soils. Inhabitants of the marine environment have to be specifically adapted to the unique environmental factors including low temperature, high pressure, high salinity and low nutrient content. The density of heterotrophic bacteria in seawater is reported to be in the range 10^5^–10^7^ per mL. Numbers and species composition vary according to the location and depth—especially in the deep sea, where the low temperatures limit the growth of microorganisms—but an average of about 10^6^ cells per mL seems to be generally accepted.^[^ ^22^, ^59^ ^]^ Whitman et al, report values of 5 × 10^5^ prokaryotes (autotrophic and heterotrophic bacteria as well as archaea) per mL for the upper ocean and 0.5 × 10^5^ prokaryotic cells per mL for water below 200 m.^[^ ^60^ ^]^ At depth below 4000 m, the frequency of microbial species decreases further due to the low temperature and low food supply. Most microorganisms of the deep sea are found on sunken organic material.^[^ ^61^ ^]^

In contrast to the rather cold marine environment, the composting process is usually carried out at a higher temperature between 58 °C and 65 °C (ISO14855‐1). The microbial species directly related to biodegradation are significantly different from those in seawater, and the number is often higher, often more than 10^9^ mL^−1^.^[^ ^62^ ^]^ Therefore, degradation profiles of biodegradable polyesters in seawater might be very different from those in soil or composting environments.

### 3.2 Seawater Degradation

We summarized and compared the performance of several common biodegradable polymers in seawater (PLA, PBAT, PBS, PHAs, and PCL, **Table** **1** lists their chemical characteristics, while **Table** **2** summarizes typical conditions for degradation in different environments). The experimental conditions including time scale, water conditions, and the sample conditions were analyzed and listed in **Table** **3**. The changes of sample morphology, mechanics strength, and molecular weight were listed as well.

**Table 1:** Comparison of mechanical properties, crystallinity, degradability, processing properties, and cost of commercial biodegradable resins with non‐degradable resins

Polymer	*T*_g_ [°C]	*T*_m_ [°C]	Tensile strength [MPa]	Elastic modulus [MPa]	Elongation at break [%]	Market share [%]	Production cost [€/ton]
LDPE	−100	98–115	8–20	300–500	100–1000	–	1150
Starch	–	–	–	–	–	38.4	400
PHB	5–10	177–182	40	700–1800	6–8	2.2	3800–6400
PHBV	0–30	100–150	10–40	600–1000	10–500		
PLA	40–70	130–180	44–65	2800–3500	10–240	25.0	2200–2600
PCL	−60	59–65	4–28	390–470	700–1000	<2.5	3000
PBS	−32	114	40–60	500	170–500	7.7	2000
PBAT	−30	110–115	25–40	65–90	500–800	24.1	1500
PGA	30–40	225–230	89	7000–8400	30	<2.5	–
PVA	58–85	150–230	28–65	30–530	50–220	–	–

**Table 2:** Comparison of the seawater environment and composting environment.^[^ ^61^, ^62^ ^]^

	Microbes [mL]	Water	Oxygen [mg L^−1^]	Salt [%]	Temperature [°C]	Pressure [atm]	pH	External force
Offshore	10^1^–10^5^	+++++	4–9	<30	≈17.4	1–20	8.0–8.7	√
Upper ocean	5 × 10^5^	+++++	4–9	≈35	≈17.4	20–1100	8.0–8.7	√
Below 200 m	5 × 10^4^	+++++	4–9	≈35	0–4	20–1100	8.0–8.7	√
Compost	>10^9^	+++++	≈310	<0.05	48–65	≈	≤7	√
Soil	10^6^–10^9^	++	≈20	<0.05	20–40	≈1	≤7	√

External force: mechanical forces from tides, waves, and other factors in natural seawater.

**Table 3:** Performance of biodegradable polyesters in seawater

Polymer	Shape/size^a,b)^	Environmental conditions	Time studied	Degradation^b)^			Ref.
				Weight loss [%]	*M*_n_ decrease [%]	Mechanical strength decrease [%]	
PLA:							
Polylactide, PLA	Spline, 180 × 10 × 4 mm	Lorient harbor, France 11–19 °C	180 days	–	Unchanged	Unchanged	^[^ ^18^ ^]^
Poly(l‐lactide), PLLA	Spline, 80 × 4 × 2 mm	Coastal Bohai Bay, China	52 weeks	2%	13%	0	^[^ ^21^ ^]^
Polylactide, PLA	Film, 12 × 12 × 0.32 mm	Artificial seawater at 25 °C	1 year	Unchanged	–	–	^[^ ^65^ ^]^
Poly(l‐lactide), PLLA	Film, 30 × 3 × 0.05 mm	Static seawater from the Pacific Ocean (Terasawa‐cho, Toyohashi, Aichi, Japan) 25 °C	10 weeks	Unchanged	Unchanged	Unchanged	^[^ ^63^ ^]^
Poly(l‐lactide), PLLA	Film, 30 × 3 × 0.05 mm	Pacific coast of the main island of Japan 19–26 °C	5 weeks	25%	Unchanged	100%	^[^ ^64^ ^]^
PHB:							
Poly(3‐hydroxybutyrate),PHB	Film, 12 × 12 × 0.32 mm	Artificial seawater at 25 °C	1 year	6%	Unchanged	–	^[^ ^65^ ^]^
Poly(3‐hydroxybutyrate), PHB	Film, 30 × 20 × 0.1 mm	Osaka port & Misaki town, Japan	6 weeks	40–100%	12–29%	–	^[^ ^22^ ^]^
Poly(3‐hydroxybutyrate) PHB	Film, 0.005 and 0.1 mm thickness	South China Sea (Vietnam)	160 days	58% for 0.005 mm film; 38% for 0.1 mm film	25.7%; for 0.005 mm film; 19.8% for 0.1 mm film	–	^[^ ^25^ ^]^
Poly(3‐hydroxybutyrate), PHB	Film, 30 × 3 × 0.05 mm	Static seawater from the Pacific Ocean (Terasawa‐cho, Toyohashi, Aichi, Japan), 25 °C	10 weeks	9%	Unchanged	40%	^[^ ^63^ ^]^
Poly(3‐hydroxybutyrate), PHB	Spline, 30 × 3 × 0.05 mm	Pacific coast of the main island of Japan 19–26 °C	5 weeks	65%	Unchanged	100%	^[^ ^64^ ^]^
Poly(3‐hydroxybutyrate) PHB	Film, 10 mg, 0.1 mm thickness	Seawater in lab (from Tokyo bay and Pacific Ocean, Japan) 25 °C	28 days	41% for bay; 23% for ocean	–	–	^[^ ^66^ ^]^
Poly(3‐hydroxybutyrate), PHB	Film, 0.15–0.19 mm thickness	Artificial seawater according to ASTM 6691	49 days	30%	–	–	^[^ ^67^ ^]^
P3HB Copolymers:							
Poly(3‐hydroxybutyrate‐*co*‐3‐hydroxyhexanoate)s PH3B‐*co*‐HH	Film, 30 × 20 × 0.1 mm	Misaki town, Japan		6 weeks	19%	–	–
Poly(3‐hydroxybutyrate‐*co*‐3‐hydroxyvalerate, P(3HB‐*co*‐3HV)	Film, 0.005 and 0.1 mm thickness	South China Sea (Vietnam)	160 days	54% for 0.005 mm film; 13% for 0.1 mm film	15.9% for 0.005 mm film; 57% for 0.1 mm film	–	^[^ ^25^ ^]^
Poly(3‐hydroxybutyrate‐*co*‐3‐hydroxyvalerate P(3HB‐*co*‐3HV) Poly(3‐hydroxybutyrate‐*co*‐4‐ hydroxybutyrate), P(3HB‐*co*‐4HB)	Fibers, 0.26 mm diameter	Jogashima, Japan.	8 week	65% for P(3HB‐*co*‐3HV), no weight loss for P(3HB‐*co*‐4HB)	27.1% for P(3HB‐*co*‐4HB)	100%	^[^ ^34^ ^]^
Poly(3‐hydroxybutyrate‐*co*‐3‐hydroxyvalerate, P(3HB‐*co*‐3HV)	Film, 10 mg, 0.1 mm thickness	Seawater in lab (from Tokyo bay and Pacific Ocean, Japan) 25 °C	28 days	100% for both bay and ocean	–	–	^[^ ^66^ ^]^
Poly(3‐hydroxybutyrate‐*co*‐4‐ hydroxybutyrate), P(3HB‐*co*‐4HB)	Film, 10 mg, 0.1 mm thickness	Seawater in lab (from Tokyo bay and Pacific Ocean, Japan) 25 °C	28 days	70% for bay; 59% for ocean	–	–	^[^ ^66^ ^]^
Poly(3‐hydroxybutyrate‐*co*‐3‐hydroxyvalerate) P(3HB‐*co*‐3HV)	Spline, 180 × 10 × 4 mm	Lorient harbour (France) 8.6–19.8 °C	360 days	8%	Unchanged	Unchanged	^[^ ^68^ ^]^
Poly(3‐hydroxybutyrate‐*co*‐3‐hydroxyvalerate) P(3HB‐*co*‐3HV)	Film, 0.115 mm thickness	Baltic Seawater	6 weeks	60%	11.8%	–	^[^ ^69^ ^]^
Poly(3‐hydroxybutyrate‐*co*‐3‐hydroxyvalerate) P(3HB‐*co*‐3HV)	Fiber, monofilament	Rausu, Toyama, and Kume, Japan. 2–10 °C, deep in 321–621 m	12 months	–	–	80–100%	^[^ ^70^ ^]^
Poly(3‐hydroxybutyrate‐*co*‐3‐hydroxyvalerate) P(3HB‐*co*‐3HV)	Film, 0.15–0.19 mm thickness	Artificial seawater according to ASTM 6691	49 days	16%	–	–	^[^ ^67^ ^]^
PCL:							
Poly(*ε*‐caprolactone), PCL	Film, 12× 12 × 0.32 mm	Artificial seawater at 25 °C	1 year	0.5%	–	–	^[^ ^65^ ^]^
Poly(*ε*‐caprolactone), PCL	Film, 30 × 20 × 0.1 mm	Misaki town, Japan	6 weeks	98%	Unchanged	–	^[^ ^22^ ^]^
Poly(*ε*‐caprolactone), PCL	Film, 30 × 3 × 0.05 mm	Static seawater from the Pacific Ocean (Terasawa‐cho, Toyohashi, Aichi, Japan), 25 °C	10 weeks	25%	Unchanged	95%	^[^ ^63^ ^]^
Poly(*ε*‐caprolactone), PCL	Film, 30 × 3 × 0.05 mm	Pacific coast of the main island of Japan 19–26 °C	5 weeks	34%	Unchanged	100%	^[^ ^64^ ^]^
Poly(*ε*‐caprolactone), PCL	Film, 10 mg, 0.1 mm thickness	Seawater in lab (from Tokyo bay and Pacific Ocean, Japan) 25 °C	28 days	100% for bay; 67% for ocean	–	–	^[^ ^66^ ^]^
Poly(*ε*‐caprolactone), PCL	Fiber, Monofilament	Rausu, Toyama, and Kume, Japan. 2–10 °C, deep in 321–621 m	12 months	–	–	80–100%	^[^ ^70^ ^]^
Poly(*ε*‐caprolactone), PCL	–	Baltic Seawater from Gdynia Harbour, 8–21 °C	2 months	100%	–	100%	^[^ ^71^ ^]^
Poly(*ε*‐caprolactone), PCL	Spline, 80 × 4 × 2 mm	Coastal Bohai Bay, China	52 weeks	30%	0.9%	20%	^[^ ^21^, ^72^ ^]^
PBS:							
Polybutylene succinate, PBS	Spline, 80 × 4 × 2 mm	Coastal Bohai Bay, China	52 weeks	2%	63%	60%	^[^ ^21^ ^]^
Polybutylene succinate, PBS	Film, 30 × 20 × 0.1 mm	Osaka port, Japan	6 weeks	2%	19%	–	^[^ ^22^ ^]^
Polybutylene succinate, PBS	Film, 10 mg, 0.1 mm thickness	Seawater in lab (from Tokyo bay and Pacific Ocean, Japan) 25 °C	28 days	2%	–	–	^[^ ^66^ ^]^
Polybutylene succinate, PBS	Fiber, Monofilament	Rausu, Toyama, and Kume, Japan. 2–10 °C, deep in 321–621 m	12 months	–	–	0–10%	^[^ ^70^ ^]^
PBAT:							
Poly(butylene adipate‐*co*‐terephthalate), PBAT	Spline, 80 × 4 × 2 mm	Coastal Bohai Bay, China	52 weeks	2%	56%	43%	^[^ ^20^, ^21^ ^]^
Poly(butylene adipate‐*co*‐terephthalate), PBAT	Film, 30 × 20 × 0.1 mm	Osaka port, Japan	6 weeks	7%	32%	–	^[^ ^22^ ^]^

^a)^Film, the sample with thickness less than 1 mm; spline, the sample with thickness equal or more than 1 mm; ^b)^ The symbol “—“represents this information was not mentioned in the document.

#### 3.2.1 Polylactide

PLA as the most common biodegradable plastic, which degrades in the compost over time, proved a dramatically reduced degradability in seawater, similar to it in pure water. Tsuji and Suzuyoshi studied the degradation properties of PLA films (0.05 mm thickness) in seawater under natural conditions and in the lab in collected seawater, that is, under static conditions. They found that the overall properties of the PLA films did not change significantly after 10 weeks in laboratory conditions. Because of the plasticization process, tensile strength and Young's modulus even increased slightly at the beginning of the experiment.^[^ ^63^ ^]^ Under natural conditions in the ocean, mechanical forces resulted in the fracture of the films after 5 weeks, resulting in higher weight loss and mechanical reduction, but GPC showed no significant changes in molar mass.^[^ ^64^ ^]^ Deroine et al. studied the degradation of PLA splines (4 mm thickness) in the Lorient harbor (France) during 6 months, no significant change in molar mass and mechanical properties was observed except some tensile strength loss.^[^ ^18^ ^]^ Compared with distilled water at room temperature, the high salinity in seawater affects the diffusion of water into the polyester, making the degradation rate in seawater even slower than that in pure water. To further predict the lifetime of PLA splines (2 mm thickness) in seawater, Wang, et al. and Agarwal et al. extended the degradation time to 1 year in natural seawater. The experiment confirmed the previous data, shown that PLA was hardly degraded in seawater.^[^ ^21^ ^]^

#### 3.2.2 Polyhydroxyalkanoates

In contrast to PLA, PHAs undergo faster hydrolysis, also in seawater.^[^ ^73^ ^]^ Already in 1992, seminal studies on the degradation of PHAs in seawater have been reported.^[^ ^34^, ^52^, ^66^, ^67^, ^68^, ^74^, ^75^ ^]^ The authors found that the degradation mechanism for PHAs in seawater followed surface erosion, as it was reported in soil and compost. However, the degradation rate of PHAs in seawater was significantly slower. As reported by Rutkowska et al., PHBV films (0.115 mm thickness), which completely degraded in compost within 6 weeks, only proved 60% weight loss when immersed in seawater after the same time. While the blend of PHBV with 60 wt% PHB resulted in a weight loss of 100% and 38% as degraded in compost and seawater, respectively.^[^ ^69^ ^]^ Volova et al. studied the degradation of PHB and P(3HB‐3HV) films in the South China Sea for 160 days and identified several PHA‐degrading strains as *Enterobacter* sp. (four strains), *Bacillus* sp. and *Gracilibacillus* sp.^[^ ^24^, ^25^ ^]^ They also demonstrated that the degradability of PHAs in seawater significantly affected by the shape and the size of the material. The study of PHB and P(3HB‐*co*‐3HV) films in seawater after 160 days proved that the weight loss of film with 0.1 mm thickness was 38% and 13%, respectively, indicating that degradation of PHB was faster than that of P(3HB‐*co*‐3HV) under the same conditions. Furthermore, the weight loss increased to 58% and 54%, respectively, when the thickness of the film was decreased to 0.005 mm. For the surface corrosion process, the size of the sample, especially the thickness and surface area determine the rate of degradation. Recent work from Laycock et al. investigated the rate of biodegradation of PHA in the marine environment and applied this to the lifetime estimation of PHA products.^[^ ^76^ ^]^ The average degradation rate of PHA in the ocean was determined as 0.04–0.09 mg day^−1^ cm^−2^. The thickness and size of the product had an important influence on the time required for its final degradation. For 0.035 mm thickness bags, the time required for complete degradation may take 25 days to 2 months, while 0.8 mm thickness bottles need 1.5 years.

#### 3.2.3 Polycaprolactone

**Figure 3:**
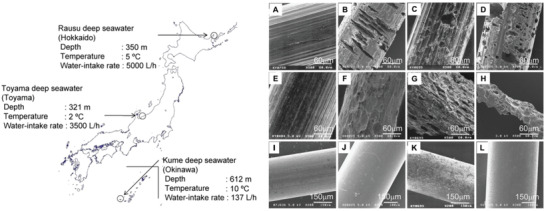
Scanning electron micrographs of A–D) PCL, E–H) PHB/V, and I–L) PBS monofilament fibers before and after soaking in deep seawaters for 12 months. (A, E, I): not soaked; (B, F, J): soaked in Rausu water; (C, G, K): soaked in Toyama water; (D, H, L): soaked in Kume water. Reproduced with permission.^[^ ^70^ ^]^ Copyright 2011, Elsevier.

Several papers report the degradation of PCL in seawater.^[^ ^64^, ^71^, ^77^ ^]^ An early work reported by Kasuya et al. studied the degradation properties of PCL in river, lake water, and seawater,^[^ ^66^ ^]^ proved that PCL can degrade in most natural waters as the degrading microorganisms are widely present in the different waters. Enoki et al. evaluated the degradation of PCL fibers after 12 months soaking in deep seawaters at Rausu, Toyama, and Kume respectively in Japan.^[^ ^70^ ^]^ With the significant mechanical decline, the surface of PCL fibers appeared heterogeneous pinholes and cracks under the action of microorganisms (see below) suggesting that significant biodegradation of the fibers occurred in the waters (**Figure** **3**). Five PCL‐degrading bacteria were screened and isolated from deep seawaters pumped up at those three locations by using a PCL granule‐containing agar medium. The isolates were found to belong to the genus *Pseudomonas*, which is known to be widely distributed in natural environments and includes several aliphatic polyester degrading bacteria, as well as two others: *Alcanivorax* and *Tenacibaculum*, which had not been reported as aliphatic polyester‐degrading bacteria. Fast degradation of PCL films with 0.1 mm thickness was reported in a laboratory test when immersed in seawater from the bay.^[^ ^66^ ^]^ The degradation properties were found to be similar to that of PHBV in the same seawater from the bay but reduced in the water from the ocean. The rate of degradation decreased as the thickness of the material increased, the 0.05 mm thickness PCL films reduced the mechanical strength by 100% and lost 30% of its original weight in natural seawater after 5 weeks.^[^ ^63^ ^]^ In our recent work, 2 mm thickness PCL splines were immersed in natural seawater located in BoHai China and a 30 wt% loss compared to its original weight after 52 weeks was measured. Analysis of the remainders with respect to size, its molecular weight, and mechanical strength indicated that a surface erosion mechanism occurred, similar to other reports from the literature.^[^ ^72^, ^74^ ^]^

#### 3.2.4 Poly(butylene Adipate Terephthalate)

PBAT is the second‐largest class of polyesters in the market. As the development and application of PBAT appeared only in recent years, research on the degradation properties of PBAT in seawater is rare. Alvarez‐Zeferino et al. studied the biodegradation of oxo‐degradable LDPE and compostable PLA/PBAT blends on marine environments through a respirometric lab test for 48 days with a marine inoculum.^[^ ^78^ ^]^ The compostable plastic exhibited a higher degree of mineralization (10%), while there was no difference between the polyolefins (2.06–2.78%), with or without the presence of pro‐oxidants or previous abiotic degradation. On the other hand, exposition to UV light promoted a higher loss of elongation at break of the oxo‐degradable plastic (>68%). Their results underline the low biodegradation rates while presenting a higher rate of loss of physical integrity. This combination of phenomena could lead to their fragmentation before significant biodegradation can occur and might promote microplastics formation. Recently, we reported the degradation of PBAT splines after 56 weeks in Tianjin BoHai Bay, China. To avoid the inaccurate weight loss and mechanical properties due to film fragility, we used a standard spline with a thickness of 2 mm. The study found that although the molecular weight and mechanics decreased to nearly half of the original, no weight loss was detected. The surface electron microscopy of the spline showed that some degraded pores were produced on the surface of the spline with the action of microorganisms, while the internal microstructure of the spline did not change significantly.^[^ ^20^ ^]^

#### 3.2.5 Polybutylene Succinate

Degradation studies of PBS in seawater are rare. In 1998, it was reported that the weight loss of 0.1 mm PBS film, with *M*_n_ of 3,300 mol g^−1^ placed in seawater taken from both bay and the ocean did not exceed 2% after 28 days.^[^ ^66^ ^]^ In 2011, Enoki et al. placed fibrillar PBS samples in deep seawaters at Rausu, Toyama, and Kume in Japan for 12 months proving a much slower degradation of PBS in these three waters compared to previously mentioned PCL and PHBV. Different from a significant loss of mechanical properties and obvious biodegradation pinholes and cracks on the surface of the fiber were observed for both PCL and PHBV, strength retention of PBS fiber decrease less than 10% after 12 months. Rough surfaces with many spots only observed in Toyama water and the surface of PBS fiber soaking in Rausu and Kume waters resulted in negligible changes (Figure 3).^[^ ^70^ ^]^

An early work of Kasuya et al. compared the degradability of eight polyesters in different types of waters by monitoring the time‐dependent changes in the biochemical oxygen demand (BOD) and weight loss of polyester film. These polyesters include the currently commercialized P(3HB), PHBV, PBS, and PCL, as well as poly(ethylene succinate) (PES), poly(ethylene adipate) (PEA), poly(butylene adipate) (PBA), which are not widely used today (**Table** **4**).^[^ ^66^ ^]^ They demonstrated that PHB and its copolymers and PCL exhibited complete weight loss and high BOD in seawater (after 28 days) while biodegradation values of PBS and poly(butylene adipate) reached ≈2% or 11%, respectively, under these conditions in seawater (ocean) after 28 days of incubation at 25 °C. Unfortunately, this work did not include PLA and PBAT.

**Table 4:** Weight‐loss biodegradability and BOD biodegradability of aliphatic polyester films in different natural waters for 28 days at 25 °C.^[^ ^66^ ^]^

Sample		Molar mass	Freshwater (river)		Freshwater (river)		Seawater (bay)		Seawater (deep sea)	
		*M*_n_10^−3^	^a)^WL [%]	^b)^BOD [%]	^a)^WL [%]	^b)^BOD [%]	^a)^WL [%]	^b)^BOD [%]	^a)^WL [%]	^b)^BOD [%]
1	P(3HB)	350	100 + 0	75 ± 16	93 ± 7	52 ± 7	41 ± 16	27 ± 10	23 ± 13	14 ± 10
2	P(2HB‐*co*‐14%3HV)	186	100 ± 0	76 ± 2	100 ± 0	71 ± 1	100 ± 0	84 ± 2	100 ± 0	78 ± 5
3	P(2HB‐*co*‐10%4HV)	223	100 ± 0	90 ± 1	74 ± 26	55 ± 17	70 ± 30	51 ± 27	59 ± 15	43 ± 14
4	Poly(*ε*‐caprolactone)	110	100 ± 0	75 ± 8	100 ± 0	77 ± 1	100 ± 0	79 ± 2	67 ± 21	56 ± 9
5	Poly(ethylene succinate)	30	100 ± 0	83 ± 2	100 ± 0	77 ± 2	2 ± 1	1 ± 1	5 ± 2	3 ± 2
6	Poly(ethylene adipate)	40	100 ± 0	70 ± 3	95 ± 5	68 ± 8	100 ± 0	65 ± 13	57 ± 14	46 ± 13
7	Poly(butylene succinate)	30	2 ± 1	3 ± 1	22 ± 14	12 ± 8	2 ± 2	1 ± 1	2 ± 3	2 ± 0
8	Poly(butylene adipate)	30	24 ± 7	20 ± 4	80 ± 13	50 ± 10	34 ± 2	20 ± 2	11 ± 10	10 ± 5

^a)^ Weight‐loss biodegradability.

^b)^ Biochemical oxygen demand (BOD).

**Figure 4:**
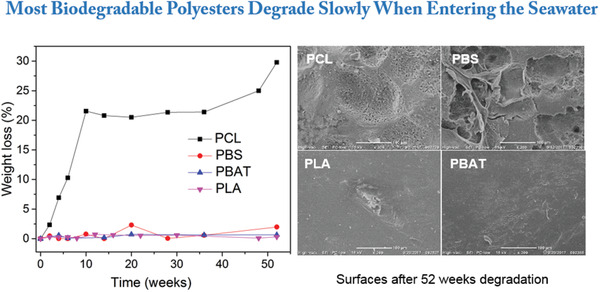
Left: Weight loss of commercial biodegradable polyesters in natural seawater during 52 weeks. Right: Scanning electron micrographs of splines soaked in natural seawater after 52 weeks. White bars show scales (Reproduced under an Open Access license.^[^ ^21^ ^]^ Copyright 2020.

The degradation performance for today's most used commercial polyesters, PCL, PLA, PBS, and PBAT splines were studied in our recent work. The spline samples with 2 mm thickness were soaked in natural seawater located at BoHai Bay, China for 52 weeks. The degradation rates of all polyester were significantly reduced compared to composting conditions. However, PCL exhibited the fastest surface erosion with a weight loss of 32% after 52 weeks. Importantly, under these comparable conditions, surface morphology, weight, molecular weight as well as the mechanical properties of PLA almost did not change after 52 weeks. Both for PBS and PBAT, the surfaces of the splines showed some roughening, which indicated a certain degree of biodegradation, however sample weight and molar mass of the samples remained relatively unchanged.^[^ ^21^ ^]^ In summary, comparison of weight loss and the changes of both molar mass and strength retention under the same experimental conditions, the degradability were sorted into PCL >> PBS > PBAT > PLA (**Figure** **4**), which corroborated well with other literature.^[^ ^65^, ^70^ ^]^

To date, only few articles have studied the degradation performance of biodegradable plastics in seawater, thus only a vague estimation on the degradability can be get through the weight loss, molecular weight or mechanical analysis of these materials in seawater within one or two years. In fact, as the degradation performance is greatly affected by the environment and the sample condition, and most of these materials degrade so slowly in seawater, that it is hard to obtain the exact degradation rate of specific materials in seawater (except for PHAs, an average rate of 0.04–0.09 mg day^−1^ cm^−2^ was estimated by Laycock et al.). The degradation performance within a longer time scale needs to be further investigated.

### 3.3 Factors Influencing Seawater‐Degradation of Polyesters

**Figure 5:**
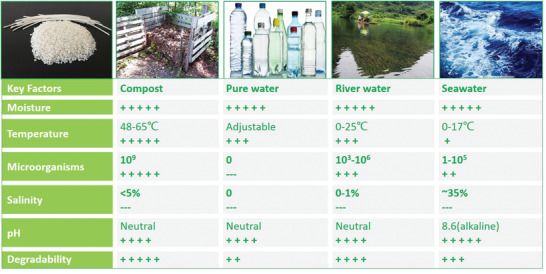
Factors affect the degradation of polyesters in different environments.

Although the decreased biodegradation rate in seawater has been reported in many studies, the fundamental reasons for this result are hardly explained. Despite strong UV light from the sea surface, mechanical forces caused by ocean currents and ocean waves in the epipelagic zone may accelerate the transition of materials from large objects to small fragments in the first step. Although pH and buffer capacity of the ocean is also dictated by the equilibrium of atmospheric CO_2_ absorption by the oceans, temperature, pressure, and diffusion at different ocean depths, the high ionic strength of seawater still make it as equivalent to a weak alkaline buffer with pH of ≈8.0–8.6, which may increase the degradation driving force compare with neutral freshwater.^[^ ^79^ ^]^ The low temperature and different microbial communities remain the most critical determinants of the degradation rate of polyesters and lead to the opposite effect. Materials that sink to deeper zones with a temperature of approximately 2 °C show dramatically lower rates of hydrolysis compared to soil and compost. Low temperature and high salinity in the marine environment lead to different microbial communities compared to soil and freshwater ecosystems, lower total amounts of microorganisms and fewer specific microbial species required for the degradation of most biodegradable materials. In this case, the second key step of biotic hydrolysis is slowed down due to the small number of active microbes. Abiotic hydrolysis always occurs, but at an even slower rate owing to the low temperature (**Figure** **5**).

PLA degrades very slowly in seawater mostly because of the lack of effective microorganisms and only slow abiotic hydrolysis occurs. Microbial degraders of PBAT and PBS are also sparse in marine ecosystems so that the whole degradation rate is slow although corrosion happened at the surface of the spline. In contrast, the relatively rapid degradation of PHAs and PCL in seawater by surface erosion can be explained by the existing of high numbers of microorganisms that can degrade these polyesters. The degradation rate of the polymers increases as the number of microorganisms increases.^[^ ^66^, ^72^, ^80^ ^]^ This conclusion is confirmed by degradation properties of PCL splines in different waters.^[^ ^72^ ^]^ The weight loss of PCL in pure water was very slow as it did not exceed 3% within 52 weeks. Similarly, molecular weights after immersing PCL in sterilized artificial seawater did not exceed a weight loss of 3%. Significant degradation only occurs in waters containing microorganisms. However, the degradation rates significantly differ depending on the types and amount of microorganisms. After 52 weeks, a weight loss of 14% was detected in static river water, while 12% weight loss was measured in laboratory static seawater. In contrast, when the PCL samples were immersed in natural seawater, a weight loss of 32% after 52 weeks was measured.

The impact of microbes can also be reflected by the performance of polyester in different zones in the ocean. Environmental factors such as temperature and microorganisms in seawater differ during the seasons of the year, at different regions and different depths, which may lead to differences in degradation performance of the same material. Consistent with the relatively low abundance of microbial populations, bioplastic degradation at the deeper‐water areas exhibited an initial lag period, after which degradation rates comparable to that at the other stations. Presumably, significant biodegradation occurred only after colonization of microorganisms on the plastic, a parameter that was dependent on the resident microbial populations. Therefore, it can be reasonably inferred that extended degradation lags would occur in open seawater where microbes are sparse. Kasuya et al. studied the degradation properties of polyester films at the seashore and in the ocean and compared it with the 28‐day degradation performance in inland river water and lake water.^[^ ^66^ ^]^ Most of the films that were degraded in lake water and river water showed a significantly decreased degradability after entering seawater, due to different corresponding species and quantities of microorganisms. Besides, it was reported that the degradation rates of polymers in the deep sea are significantly lower compared to bay waters, duo to different numbers and types of microorganisms (Table 4). Sekiguchi et al. evaluated the degradation of PCL, PHAs, and PBS in deep seawaters at 3 different locations (Rausu, Toyama, and Kume, Japan). They were able to prove region‐dependent degradation profiles of the same polymer.^[^ ^70^ ^]^ As biodegradable plastics typically have a higher density than seawater, they sink to deeper water with a lower number of microorganisms. Therefore, it is rational that a plastic bag with fast biodegradation kinietics on the waterfront may not degrade for a long time after entering the ocean or sinking into the deep sea.

## 4 Acceleration of Biodegradation Rates in Seawater

To ensure degradation of polymeric materials in seawater, different strategies have been reported: Either new polymers with a selective seawater‐degradation profile need to be designed or the degradation rates of other degradable polymers need to be accelerated by blending or chemical modifications. Due to the low temperature and lack of suitable microorganisms in seawater, the hydrolysis process as the speed determining step is greatly slowed down and hinders the next step of assimilation by microbes. Therefore, the key to promoting the biodegradation of polyester in seawater is to accelerate the hydrolysis process, in which the high molecular polymer is turned into small soluble oligomers or monomers.

### 4.1 Promising Seawater‐Degradable Polymers without the Need for Blending or Modification

**Scheme 2:**
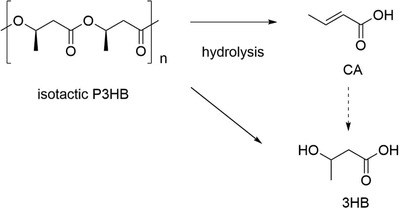
Hydrolysis of P3HB to 3HB and CA.

There are some seawater‐degradable polymers, for which microbial degraders are widely distributed in marine ecosystems. Polymeric materials based on starch or cellulose are readily degraded in seawater as microbial degraders exist in different natural environments. However, in most cases, these polymers are used as fillers and not as the main component of the material.^[^ ^81^, ^82^ ^]^ Besides, some polyesters, such as PCL and PHAs have been reported to degrade in seawater, although their degradation rates in seawater are significantly slower than that in the soil. Also new aspects of chemical routes to PHB should be mentioned here: besides bacterial poly[(R)‐3‐hydroxybutyrate], P[(R)‐3HB], a perfectly stereoregular, pure isotactic crystalline thermoplastic material,^[^ ^83^ ^]^ also ROP of cyclic lactones (4‐membered *β*‐butyrolactone^[^ ^84^ ^]^ or more recently eight‐membered cyclic diolide^[^ ^85^ ^]^) can be conducted to obtain P3HBs with adjustable tacticity and properties, which also control the degradation rates.^[^ ^85^ ^]^ More recently, in the context of chemical recycling, for the abiotic hydrolysis of poly[(R)‐3HB] under acidic or basic conditions, Yu and coworkers reported the formation of two monomeric hydrolytic products, 3HB and CA (**Scheme** **2**).^[^ ^86^ ^]^ 3HB and CA were detected as major hydrolytic products from alkaline hydrolysis, while poly[(R)‐3HB] was tolerant to hydrolysis under moderate acidic conditions. In contrast, poly[(R)‐3HB] was completely degraded into 2% of 3HB and 90% of CA in concentrated acid, during which 3HB was dehydrated to CA. Accumulation of two monomeric products, 3HB and CA, was in agreement with the mass loss of P3HB films, indicating a sequential degradation from the ends of P3HB polymers and oligomers. Such studies demonstrate the potential of P3HB as suitable seawater‐degradable polymer also by synthetic approaches; its degradation product 3HB can also be metabolized.

**Figure 6:**
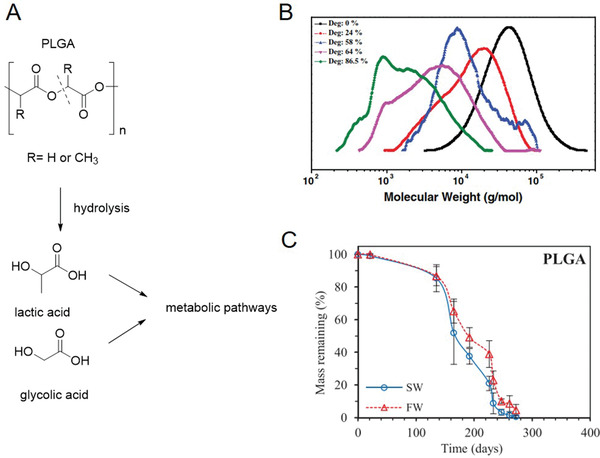
Structure of A) PLGA and degradation products; B) Gel permeation chromatography, and C) weight loss analyses of PLGA showing degradation in freshwater (FW) and seawater (SW) at different time points. B,C) Adapted under the terms of the CC‐BY 3.0 license.^[^ ^65^ ^]^ Copyright 2017. The Authors, published by Wiley‐VCH.

Furthermore, other polyesters with quick abiotic hydrolysis rates, such as PGA and PLGA can be applied in seawater‐degradable materials. PGA which synthesized by the ROP of glycolide (GA) is the simplest of aliphatic polyester with poor solubility, high melting temperature (*T*_m_) (225–230 °C) and great mechanical strength (Young's modulus *E* = 12.8 GPa).^[^ ^87^ ^]^ PGA hydrolyzes in pure water with dramatic mass loss within 5 months and loss of tensile strength after 1–2 months. Random copolymerization of GA units within LA segments disrupts the regularity of the polymer, and decreases the crystallinity of copolymers PLGA. Thus, the amorphous copolymer PLGA shows improved toughness and solubility as well as an accelerated and adjustable biodegradability caused by the more available ester bonds.^[^ ^56^, ^88^ ^]^ Agarwal et al. studied the degradation of PLGA in both artificial seawater and freshwater at 25 °C under fluorescence light (16 h light and 8 h dark) for 1 year.^[^ ^65^ ^]^ PLGA showed 100% degradation in seawater (SW) after 270 days as determined by time‐dependent weight loss, and change in molar mass; similar results were obtained for freshwater (FW, **Figure** **6**). While the initial PLGA eluated with a unimodal molar mass curve in GPC (Figure 6B). PLGA incubated in FW or SW exhibited broadening in GPC for partially degraded samples indicating chain scission and hydrolysis. The decrease in molar mass hinted for bulk degradation.^[^ ^65^ ^]^ The amorphous nature of the polymer might be responsible for the faster hydrolysis and complete degradation of PLGA, facilitating the diffusion of water throughout the bulk. Although the specific degradation process and the final product of PLGA in seawater has not yet been studied, the quick biotic hydrolysis and the produced small molecular oligomers will increase the chance of the assimilation by microbes. It should be noted that commercial PGA and PLGA are synthesized by ring‐opening polymerization from high‐cost LA and GA intermediates to ensure the high molecular weight, narrow molecular weight distribution, high purity and low by‐product for medical requirements. The high costs limit their use in other than biomedical applications.^[^ ^89^ ^]^ Polyethylene terephthalate (PET), one of the most important synthetic polymers used today is also a promising seawater‐degradable polymer in seawater. While efficient PETase was artificially produced,^[^ ^90^ ^]^ a few species of bacteria and fungi in marine have been described as capable of partially degrading PET to oligomers or even monomers. However, it is noteworthy that all known PET hydrolases have relatively low turnover rates.^[^ ^91^ ^]^ Also new polyesters, which might undergo seawater‐degradation, can be potential replacement for the slowly degrading PET. In a recent study, copolymers based on *γ*‐butyrolactone and *trans*‐hexahydrophthalide were prepared, which exhibited comparable barrier and mechanical properties to petroleum‐based PET and superior to biobased PLLA.^[^ ^92^ ^]^ Such copolymers could be used in packaging applications with a closed loop lifecycle through either their degradability or chemical recyclability; however, to date‐ no seawater degradation has been reported.

**Scheme 3:**
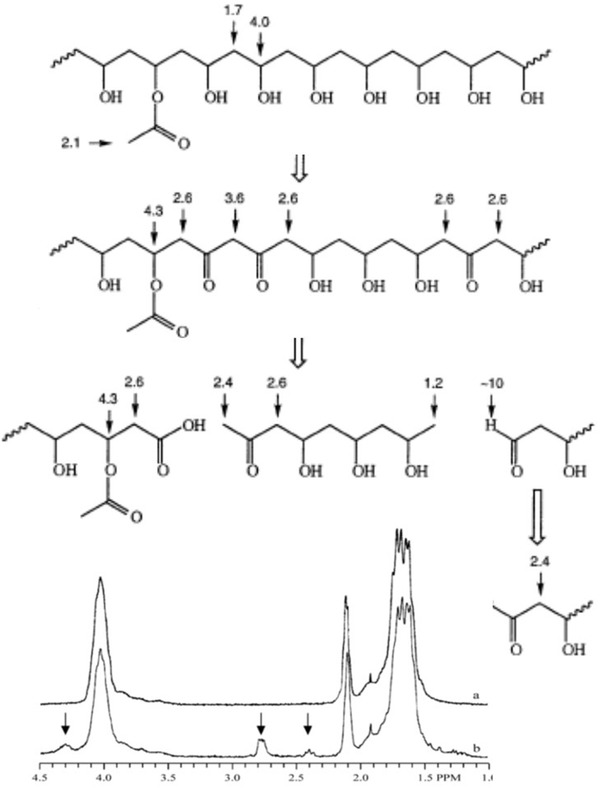
Schematic representation of the possible pathways of the oxidative enzymatic degradation of PVA and ^1^H NMR chemical shifts of the different groups. Adapted with permission.^[^ ^100^ ^]^ Copyright 2002, Elsevier.

Besides hydrophobic polymers, another class of promising seawater‐degradable polymers are water‐soluble polymers, for example, poly(vinyl alcohol) (PVA) or poly(ethylene glycol) (PEG). PVA is produced on an industrial scale by hydrolysis (methanolysis) of polyvinyl acetate. In general, the water‐solubility of PVA is highly dependent on its alcoholysis degree and the molecular weight. Different grades of PVA with varied mechanical properties and water solubility have found their applications in many fields such as s food packaging, coating, textile, cosmetics, and paper.^[^ ^93^, ^94^, ^95^, ^96^, ^97^ ^]^ Although PVA can be biodegraded under both aerobic and anaerobic conditions and a variety of micro‐organisms (e.g., members of the genera *Pseudomonas, Sphingomonas* and *Sphingopyxis*), the distribution and number of microorganisms with the ability to degrade PVA in seawater are lower compared to those that can degrade aliphatic polyesters.^[^ ^98^ ^]^ Microorganisms capable of degrading PVA are not ubiquitous but are distributed in specific environments such as wastewater especially discharged from textile and paper mills containing PVA (**Scheme** **3** shows a possible degradation pathway for PVA including NMR shifts of detected degradation products).^[^ ^93^, ^99^, ^100^ ^]^ Nogi et al. searched for PVA‐degrading microorganisms in seawater and isolated new strains of the genus *Thalassospira*.^[^ ^101^ ^]^ The genus *Thalassospira* comprises Gram‐negative, halophilic bacteria of the family *Rhodospirillaceae* of the class *Alphaproteobacteria* and includes polycyclic aromatic hydrocarbon‐degrading species that are distributed in oceans, waste‐oil pools, and petroleum‐contaminated seawater. Although microorganisms being able to assimilate PVA are rarely found in marine environments, there is still a chance of PVA degradation in these specific environments. In contrast, for ethylene vinyl alcohol copolymers (EVOH, 38 mol% ethylene) weight loss and CO_2_ evolution measurements were conducted in respirometric tests carried out in the presence of marine sediments and selected marine microorganisms.^[^ ^102^ ^]^ No significant degradation was reported, however, a marine actinomycete was found, which was able to grow in the presence of EVOH as single carbon source (at a rate too slow to be detected by the respirometric measurement).

**Scheme 4:**
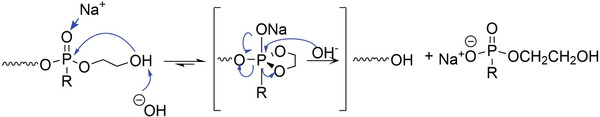
Hydrolysis mechanism of polyphosphoesters by backbiting under neutral or basic conditions. Adaptedwith permission.^[^ ^106^ ^]^ Copyright 2018, Elsevier.

PEG is used in many applications, for example, surfactants, creams, food additives, biomedical applications.^[^ ^103^ ^]^ Bernhard et al. reported the biodegradation of PEG in different waters (inocula from municipal wastewater and seawater aquarium filters).^[^ ^104^ ^]^ The authors found a distinct molar mass dependency on the biodegradation of PEG in waters, for example, PEG (*M*_n_ = 920 g mol^−1^) degraded completely after ca 1 month, while the higher molar mass PEGs degraded slower (PEG with *M*_n_ of ≈50 kg mol^−1^, proved less than 20% biodegradation after 130 d). Concerning water‐soluble polymers, Wurm's lab recently studied a variety of polyphosphoesters (PPEs). It was found that their degradation rates can be tailored by the chemical structure, that is, polyphosphonates degraded much faster (days to weeks) under basic conditions (pH = 8, similar to seawater),^[^ ^105^ ^]^ while polyphosphates degraded very slowly under these conditions. We believe that the increased degradation rates of polyphosphonates are related to the degradation mechanism by backbiting (**Scheme** **4**) and are probably caused by the electron density of the central phosphorus and the different tendency to form the 5‐membered cyclic intermediate.^[^ ^106^ ^]^ Another report proved the seawater‐degradation of polyphosphonates, which were studied as kinetic hydrate inhibitor polymers (biodegradation of about 31% using the marine OECD 306 test protocol was reported).^[^ ^107^ ^]^

Also, other water‐soluble polymers have been studied for their seawater degradability, for example, polyoxazolines (seawater biodegradation studies according to the OECD306 procedure indicated their poor biodegradability (<20% in 28 days))^[^ ^108^ ^]^ and poly(vinyl pyrrolidone) (almost no degradation in sewage sludge was reported).^[^ ^109^ ^]^

### 4.2 Seawater Degradable Blends Containing Degradation Promoters

#### 4.2.1 Blends with Readily Degradable Polymers

To increase degradation rates in seawater, the addition of readily biodegradable fillers can help to accelerate the degradation of the polymer matrix. This strategy was widely applied and investigated when some products are required to give a certain degree of biodegradability in soil or compost.^[^ ^110^ ^]^ Starch and cellulose (or derivatives) have been used in this approach, as they are cheap, widely available, exhibit good compatibility and can be readily degraded by yeast, fungi, and various bacteria.^[^ ^38^, ^82^, ^111^ ^]^ Rutkowska et al. investigated the effect of blending starch (5% and 8%) into polyethylene on the degradation in the Baltic Sea at Nordic Wharf of Gdynia harbor.^[^ ^112^ ^]^ It was expected that the degradation process of PE should be sped up through microbiological consumption of the starch particles, producing a higher surface/volume ratio of the polyethylene matrix. The experiment took place for 20 months and it was found that in natural seawater the enzymatic hydrolysis of starch occurred and demonstrated by clear erosion of the surface and weight loss, but the remaining PE with the increased surface area did not show increased weight loss compared to bulk PE control (less than 1% weight loss was determined). This result demonstrated that blending polyethylene with starch (5 and 8%) did not increase the microbial degradation of PE bulk in seawater under natural conditions.

Guzman‐Sielicka et al. tested the degradation of different proportions of PLA‐starch‐CaCO_3_‐glycerol blends in seawater under laboratory conditions.^[^ ^113^ ^]^ After 4 weeks of incubation, all samples were fragmented with a maximum weight loss of 73%, similar to the removal of the additive amount (72%). The degradation was still not completed even after 4 months under these conditions, the degradation of PBAT and its composites containing starch (PBAT‐starch) proved similar results.^[^ ^20^ ^]^ The test splines were immersed in static river water, static seawater, natural seawater, static sterilized distilled water, static sterilized seawater, and static sterilized lab‐prepared seawater for 56 weeks. The results underlined that the pure PBAT degraded very slowly in all water samples with a maximum weight loss of only 4.7% after 56 weeks. In contrast, PBAT‐starch composites showed significantly higher weight loss in microbe‐containing river water and seawater. However, degradation occurred almost exclusively in the starch fraction, the degree of degradation depended largely on the type and abundance of microorganisms in the water bodies. The rate of weight loss in river water with the highest number of bacteria was 32%, and in seawater only 3.3%.

Recently, BioLogiQ, a US biofuels manufacturer, announced the successful development of a new thermoplastic polymer, so‐called NuPlastiQ, which is based on potato starch. NuPlastiQ is mixed with PBAT and is called a NuPlastiQ MB biopolymer. The new product is said to undergo marine biodegradation within 1 year. However, the degradation properties, final products, and degradation properties of different shapes are to be further evaluated.^[^ ^114^ ^]^

The degradation rates of similar starch blends increased dramatically when the polymer matrix itself proved to be degradable in seawater.^[^ ^115^ ^]^ Imam et al. investigated the degradation of PHBV blended with corn starch at four different places in coastal water southwest of Puerto Rico for 1 year.^[^ ^75^ ^]^ Microbial enumeration at the four stations revealed considerable flux in the populations over the year. However, in general, the overall population densities of 870 CFU mL^−1^ at the deeper‐water station were 1–2 orders of magnitude less than that at the other stations. Starch degraders were 10 to 50 folds more prevalent than PHBV degraders at all of the stations. Biphasic weight loss was observed for the starch‐PHBV blends at all investigated places, as both starch and PHBV are known to undergo biodegradation in seawater. However, the degradation rates of the polymer blend, as determined by weight loss and deterioration of tensile properties, correlated with the amount of starch present (100% starch >50% starch > 30% starch > 100% PHBV). The additional incorporation of PEG into the mixture slightly retarded the rate of degradation, probably as PEG enhanced the adherence of the starch granules to PHBV in the blends. The weight loss rate of starch from the 100% starch samples was about 2% day^−1^, while the loss rate of PHBV from the 100% PHBV samples was about 0.1% day^−1^. A predictive mathematical model for loss of individual polymers from a 30% starch‐70% PHBV formulation was developed and experimentally validated. The model showed that PHBV degradation was delayed 50 days until more than 80% of the starch was consumed and predicted that starch and PHBV in the blend had half‐lives of 19 and 158 days, respectively.

Although degradation in seawater of some starch‐polyester blends increased compared with the pure polyesters, the overall degradation rates are still much slower compared to degradation in soil and compost. As the polyester matrices exhibit generally a low seawater‐degradability, the weight loss of the composite after immersing in seawater is mainly due to weight loss of the fillers. Despite this, the removal of the fillers does result in an accelerated loss of the mechanical properties of the material and speed up their fragmentation process. For biodegradable polyester matrix such as PHB, that supposed to be helpful to accelerate their end of life by microbe; for non‐degradable polymers such as PE that may lead to their fragmentation before significant biodegradation can occur and might accelerate the generation of microplastics.

#### 4.2.2 Water‐Soluble Degradation Promoters

PVA was blended with readily degradable substances such as biopolymers or biodegradable polyesters to investigate the promotion of the degradation process.^[^ ^93^, ^95^, ^96^, ^116^ ^]^ Starch (or thermoplastic starch (TPS))‐PVA‐blends are of particular interest due to the excellent compatibility of these components.^[^ ^117^ ^]^ TPS and PVA can be blended at different ratios to tailor the mechanical properties and degradability of the material for a variety of applications.^[^ ^95^, ^97^, ^118^, ^119^ ^]^ Generally, the rate and extent of biodegradation of the blends were estimated from the biodegradation behavior of the individual components. However, deviations have been reported as the degradation mechanisms can interact with each other, for example, the less degradable phase can decrease (or inhibit) the access of water into the other (normally more degradable) component. Therefore, the rate and extent of degradation of the blend may not be directly predictable from the extent and rate of degradation of the components. A systematic study on the anaerobic degradability of a series of TPS‐PVA‐ blends was performed to determine their fate upon disposal in either anaerobic digesters or bioreactor landfills.^[^ ^119^ ^]^ It was found that the presence of starch in the mixture positively influenced the rate of degradation of PVA solely; concurrently, a significant reduction was witnessed in the overall period of degradation. According to these observations, starch can be considered more than just a filler in a polymer mixture, as it even contributes to inducing the biodegradation of PVA.

When blended with polyesters that exhibit faster biodegradation rates, for example, PHB, the degradation of both components becomes relatively independent from each other since water uptake rates are similar and different microorganisms can degrade the components of the polymer blend. PVA also enhanced the mineralization rate of the PHB fraction in the blends.^[^ ^120^ ^]^ The accelerating effect of the PVA seemed to be related to processing and the lowering of PHB crystallinity in polyester‐rich blends.^[^ ^120^, ^121^ ^]^ A preliminary investigation of the influence of PVA on the biodegradation of PCL in the PVA/PCL blend films was conducted by BOD measurements.^[^ ^122^ ^]^ The experiment was carried out in axenic cultures of a specific PCL assimilating actinomycete that was isolated from the compost deriving from the organic fraction of household waste. The biodegradation of pure PCL film was completely suppressed when the cultures were supplemented with PVA, even when PCL mineralization was already started. It appeared that PVA tended to strongly adsorb on the PCL surface when it was added to the aqueous culture medium, caused a change in the surface properties of PCL films which substantially depressed the hydrophobicity required for guaranteeing the accessibility of the polymer bulk to esterase enzymes and strongly inhibited their propensity toward biodegradation.

The above studies are based on PVA blends in compost, certain bacterial culture or other freshwater bodies, the degradation properties of PVA blends in seawater may differ from them according to the huge difference in environmental factors. Unfortunately, there is only very little research in this area. Raghul et al. reported the biodegradation of PVA/LLDPE plastic film by a consortium of marine benthic vibrios. Marine bacteria, *Vibrio alginolyticus* and *Vibrio parahemolyticus* from sediments were evaluated for their ability as a consortium to degrade blends of poly(vinyl alcohol)/LLDPE films over 15 weeks (shaken at 120 rpm at 37 °C).^[^ ^123^ ^]^ The authors reported ≈20% decrease in tensile strength, when 25% and 30% PVA were blended into the composite. Visualization by SEM proved the formation of cracks on the surface of the films after 15 weeks.

**Figure 7:**
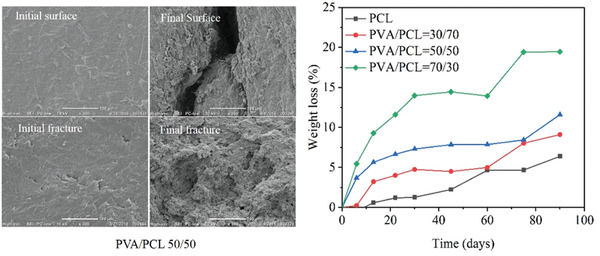
Scanning electron micrographs of PVA/PCL 50/50 blends before and after degradation (left) and time dependent weight loss (right) of PVA/PCL splines soaked in natural seawater after 3 months. Reproduced with permission.^[^ ^124^ ^]^ Copyright 2019, Elsevier.

In our recent work, PVA/PCL blends were immersed in natural seawater for three months. Both quick dissolution of PVA and biodegradation of PCL was observed. For the PVA/PCL blends in seawater, a much higher weight loss was achieved compared with that of pure PCL (**Figure** **7**). As investigated PVA can be used as an effective hydrolysis accelerator for the PCL matrix. The channels left by the quick dissolution of PVA facilitate the entry of water and microorganisms into the materials to contact the PCL, thereby promoting the biodegradation process of the PCL matrix itself.^[^ ^124^ ^]^ On the one hand, the biodegradable material is given a certain degree of water solubility, which makes the material dissolve or disintegrate quickly, achieving the loss of structure and performance in a short and controllable time. On the other hand, the water solubility of the material increases the probability of the material contacting with water and microorganisms, thus promoting the hydrolysis process and achieving rapid degradation in seawater.

#### 4.2.3 Easy Hydrolysis Degradation Accelerators

By blending biodegradable starch or water‐soluble PVA into polyesters, the vanishing soluble and quick degradable phase increase the surface area and the chance for water absorbance and microorganisms thus may promote available biodegradable process. While the mechanical properties of the polyester matrix hardly changed in the initial phase of the degradation process (especially for the high molecular weight), the overall decomposition process might be still relatively slow. To further accelerate the degradation process, it is desirable to introduce readily abiotic hydrolyzable components as additional degradation promoters. These promoters can be hydrolyzed even in an abiotic environment and produce acidic intermediate and further accelerate the degradation of the polyester matrix.^[^ ^87^, ^125^ ^]^ The blend can undergo more facile main‐chain hydrolysis even under abiotic conditions. Thus enzymatic hydrolysis is likely still feasible, but not necessary for the initial degradation steps that convert high molecular weight polymer to oligomers.

Polyoxalates are quickly hydrolyzed in water, their self‐hydrolysis process is not restricted by microorganisms and can release catalytic acid intermediates.^[^ ^126^ ^]^ Yoshikawa S. et al. proposed to blend the easily hydrolyzed polyvinyl oxalate with the biodegradable resin PLA. Since polyvinyl oxalate is easily hydrolyzed as an ester decomposition accelerator, the hydrolysis of the polyvinyl oxalate phase occurs firstly in water, and the acid is continuously released during the hydrolysis. The released acid accelerates the hydrolysis process of PLA. Besides, since a phase in the blend is hydrolyzed to cause cracking in the body, water and microorganisms are more likely to enter the inside of the body, so that decomposition of the biodegradable resin is remarkably promoted.^[^ ^127^ ^]^ This kind of hydrolyzable polyoxalates and its blends are useful as a dispersion for extracting underground resources by dispersing them in an aqueous medium.

Due to the fast in vivo hydrolysis and excellent biocompatibility, PGA or PLGA blended fibers and micro‐ and nanoparticles with PCL, PLA, collagen, and chitosan have been prepared and investigated for many years for medical research and applications, while studies and progress of this two kinds of polyester blends in other applications are very limited. Yoshikawa S. et al. proposes to use PGA as a hydrolysis accelerator for poorly hydrolyzable PLA. PGA is hydrolyzed upon contact with water and release acid which acts as a self‐catalyst for promoting decomposition of the poorly hydrolyzable PLA. Sodium carbonate was introduced to accelerate the hydrolysis of PGA and PLA. As a result, the hydrolysis promoting function by PGA is exhibited in a short time, that is, the initial speed at which the biodegradable resin is decomposed is remarkably improved. In this case, the hydrolysis rate of PLA/PGA blend is increased to 3.7 times that of PLA. However, the compatibility between PGA and PLA is low, which limits the mechanical properties and transparency of the material.^[^ ^128^ ^]^ For further improvement, they proposed to replace the above‐mentioned ester decomposition accelerator PGA with a block copolymer or random copolymer PLGA having both a relatively stable PLA and a readily hydrolyzable segment PGA in the main chain. Since the PLA fragment has good compatibility with the PLA resin matrix, the compatibility of the blend was remarkably improved and the transparency was increased. Moreover, the introduction of PLGA in the blend modifies the brittle PLA and increases the overall toughness of the material. The degradation rate of PLGA is faster than that of PGA, and the degradation properties of the material can be regulated by adjusting the ratio of GA and LA and the ratio of PLGA in the overall blend.^[^ ^129^ ^]^ This kind of hydrolyzable blends was also designed for use as a dispersion for extracting underground resources.

### 4.3 New Design of Seawater‐Degradable Polymers

A small group change in the polymer chain may cause a change of crystallinity, hydrophilicity and other properties. These changes may also affect the interaction with microorganisms and the degradation rates. So, there is a great potential for polymer chemists to design new seawater‐degradable polymers or by modifications/blending of current materials. The major challenge is to make sure that the properties of the material still are suitable for the desired application, for example, mechanical strength, heat resistance, a certain degree of stability under wet conditions, and to ensure microbial degradation after immersing into seawater.

**Figure 8:**
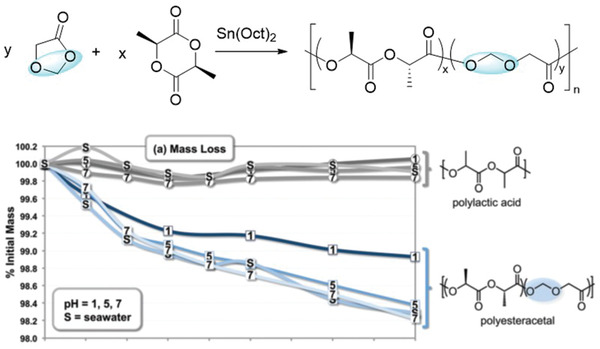
Seawater‐degradable polyesteracetal by Miller et al. showing the mass loss compared to PLA. Reproduced with permission.^[^ ^130^ ^]^ Copyright 2014, The Royal Society of Chemistry.

Martin et al. installed acetal linkages into PLA's main chain via ring‐opening copolymerization of lactide and 1,3‐dioxolan‐4‐one (**Figure** **8**).^[^ ^130^ ^]^ The polyesteracetal were designed to have thermal properties similar to those of the PLA homopolymer, yet underwent more facile main‐chain hydrolysis under abiotic conditions. The results showed that the presence of the acetal group, even at just 4 mol% abundance, substantially altered the properties and behavior of the PLA. The glass transition temperatures after acetal incorporations increased in the range of 4 mol% to 19 mol%. Facile hydrolysis of the polyesteracetal was observed in aqueous media over 45 days, including pH = 1, pH = 5, pH = 7 (distilled water), and seawater (pH = 7.5). Mass loss, molecular weight loss, in addition to surface erosion were observed for the polyesteracetal, while pure PLA showed no measurable change under these conditions.

Storey et al. reported degradable thermoplastic polyurethane (TPU) elastomers based on PBA and PLGA mixture chain‐extended by di‐cyclohexyl methane‐4, 40‐diisocyanate (H_12_MDI).^[^ ^131^ ^]^ Hydrolytic degradation tests of 1 mm thickness films in the static seawater in the lab showed enhanced degradation compared to those TPUs with only PBA as the soft segment. The latter compositions remained essentially unchanged throughout the test. As the PBA‐PLGA weight ratio increased from 100/0 to 75/25 to 50/50, the weight loss of the material in the 37 °C seawater for 22 weeks increased from 0 to 25% to 45%. Molecular weights of TPUs containing degradable polyols were lower than those derived from 100% PBA polyol. The change of the hard segment chain extender has no significant effect on the degradation performance.

**Figure 9:**
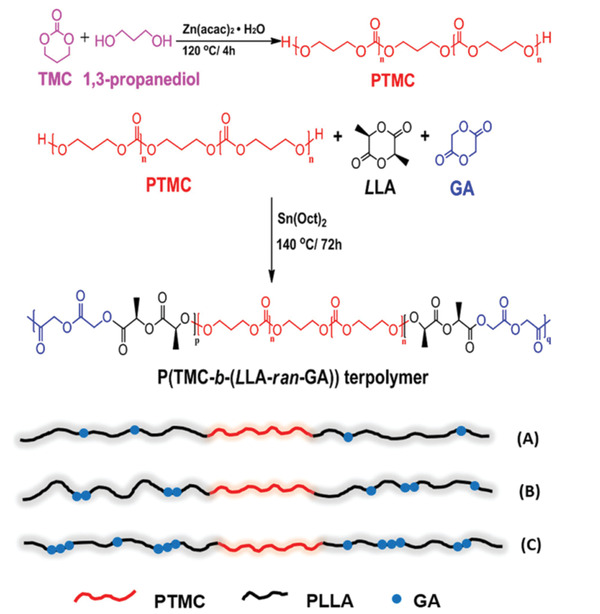
Polycarbonate‐polyester terpolymer with LA and GA segments by Fan et al. showing accelerated degradation in phosphate buffer solution compared to PLA. Reproduced with permission.^[^ ^132^ ^]^ Copyright 2018, Wiley.

Fan et al. reported a series of modified polycarbonate‐polyester terpolymers, consisting of PTMC as a polycarbonate segment attached to random copolymers of l‐lactide (LLA) and glycolide (GA) by a statistical copolymerization (**Figure** **9**).^[^ ^132^ ^]^ The terpolymers with *M*_n_ of ≈5.0 × 10^4^ g mol^−1^ and molar mass dispersity of *Ð* = 1.7 were synthesized by a two‐step ring‐opening polymerization. It showed that GA units have a significant effect on the thermal and crystallization behaviors, mechanical properties, and the biodegradability of terpolymers. The toughness of materials was improved and in vitro degradation was accelerated. Meanwhile, compared to random PLLA‐TMC‐GA terpolymer obtained by statistical copolymerization, which reported in their earlier work,^[^ ^133^ ^]^ P(TMC‐*b*‐(LLA‐ran‐GA)) terpolymer exhibited better mechanical properties.

**Scheme 5:**
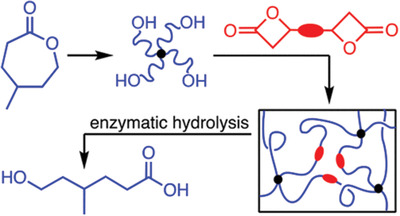
Preparation of a hydrolysable polyester elastomer by De Hoe et al. that has the potential to degrade quickly in seawater. Reproduced with permission.^[^ ^134^ ^]^ Copyright 2018, American Chemical Society.

Recently, De Hoe et al. prepared chemically cross‐linked elastomers using a novel bis(*β*‐lactone) cross‐linker and star‐shaped, hydroxyl‐terminated poly(*γ*‐methyl‐*ε*‐caprolactone) (**Scheme** **5**).^[^ ^134^ ^]^ Using model compounds, they determined that the bis(*β*‐lactone) cross‐linker undergoes acyl bond cleavage to afford *β*‐hydroxyesters at the junctions. The mechanical properties of the cross‐linked materials were competitive with a conventional rubber band. Furthermore, the elastomers demonstrated high thermal stability and a low glass transition (−50 °C), indicating a wide range of use temperatures. The polyester networks were proven to undergo enzymatic hydrolysis and they readily hydrolyzed at neutral pH and temperatures (2–40 °C) achieving complete hydrolysis. These materials might be an alternative to conventional elastomers, however, might also be seawater degradable.

In summary, copolymerization is an efficient method to construct seawater‐degradable polymers. Furthermore, the architecture and composition of materials can be easily tailored to control their properties. However, copolymerization behavior (i.e reactivity ratios of the comonomers) was not studied in detail, which might cause the formation of gradient or block‐like structures. The polymer microstructure has a strong effect on the degradation behavior and the molar mass of the resulting fragments, which shall be conducted in future works for copolymers with increased seawater‐degradability. In addition, these examples rely on the acceleration of hydrolysis in seawater, which might limit the application range of the (co)polymers. A selective and orthogonal stimulus for increasing the sweater‐degradation might be envisioned in future generations of this strategy. Also, detailed studied regarding the degradation mechanisms and the ecological effects of intermediates and final degradation products are still unknown and need further investigation.

## 5 Criteria and Methods to Evaluate Seawater Degradation of Polymers

Besides designing seawater‐degradable materials, it is also important to establish and refine standards that are used to study their degradation and to ensure comparability of the results. Specifically, the standards need to specify the requirements (e.g., conditions for degradation tests) for identifying polymer products that might even be labeled as “seawater‐degradable.” Different properties refer to the degradation of polymers and commonly used for characterization of polymers in (sea)water are summarized in **Table** **5**.

**Table 5:** Polymer properties changing during the degradation

	Weight loss	Molar mass Loss	Mechanical strength loss	Biochemical oxygen demand (BOD)	CO_2_ release
Fragmentation	Yes	Not necessarily	Yes	Not necessarily	Not necessarily
Hydrolysis	Yes	Yes	Yes	Not necessarily	Not necessarily
Biodegradation	Yes	Not necessarily	Yes	Yes	Yes
Dissolution	Yes	No	Yes	No	No

Present articles mostly testify the degradation performance of the polymer by monitoring their loss of weight, molar mass, and mechanical strength, and so forth.^[^ ^135^ ^]^ Measuring the weight loss of specimen is one of the most commonly applied tests for biodegradability. Such gravimetric measurement can be falsified by several factors: 1) Loss of weight can be caused by washing out of soluble additives or impurities. 2) Weight loss might occur during the degradation experiment in natural waters, which can lead to higher weight‐loss data. 3) The degradation products might be insoluble in water, which might lead to changed kinetics or a lower weight loss data. Another most common method relies on the change of mechanical and rheological properties, for example, tensile strength, elongation at break, and elasticity. This is the most obscure method of degradation characterization, because the mechanical properties of plastics, especially the elongation at break is very sensitive to the environment, and many factors can cause this phenomenon. For example, a slight decrease in molecular weight, partial dissolution, and the like.

The degradation process will inevitably lead to the shortening of polymer chains, so molar mass information would be to most detailed way to assess the degradation of a material. A relatively robust technique is GPC by comparing apparent molar masses and distributions from the original sample with the samples after a certain degradation time. During specific analysis the degradation mechanism needs to be considered: for a surface‐eroding sample, such as PCL and PHAs, GPC data of the residual sample will detect only little changes of the molar mass as the bulk of the sample remains intact. However, this method cannot effectively distinguish the abiotic hydrolysis process from the biodegradation process.

The most direct evidence of a biodegradation process is the observed release of carbon dioxide, the degradation product. This process is usually performed under aerobic conditions, so it can also be indirectly characterized by the oxygen consumption (biochemical oxygen demand) of microorganisms. A similar test of biodegradability of polymer in compost and/or soil is described in ISO14855‐1‐2012 and also OECD 306. The test method is conducted under controlled laboratory conditions. The sample is crushed and added to compost container under 58 °C, with cellulose as a reference. During the test, the carbon dioxide generated from the test container and the blank container is continuously monitored, and the generated cumulative amount of carbon dioxide is recorded. The biodegradation percentage of the test material is the ratio of the amount of CO_2_ produced in the test to the amount of carbon dioxide that can be theoretically produced.

ASTM‐D7081‐05 establishes a standard specification for plastic products and materials that will biodegrade satisfactorily in the marine environment. It stipulates that the biodegradable materials in the seawater must meet three requirements at the same time: disintegrate during marine degradation release carbon dioxide and have no adverse environmental impacts. While this standard has been repealed in 2014. ASTM‐D6691‐2009 offers the standard test method to assess the rate and degree of aerobic biodegradation of plastics exposed to marine microorganisms. Using a similar principle to compost degradation test method, aerobic biodegradation is determined by measuring the amount of CO_2_ produced during the exposure. The biggest difference is that the degradation test temperature is lower, 30 ± 2 °C, and the degradation medium is changed from compost to seawater. Here, the tested seawater can be either obtained from a natural seawater sample (with added inorganic nutrients) or prepared by adding various isolated marine microorganisms to the synthetic sea salt solution. This is currently the only active standard specification for the biodegradation testing method in seawater. It is worth noting that the degradation process is carried out at 30 °C, a temperature higher than in seawater and lower than in compost so that the test results only give relative degradation rates. Further from the perspective of microorganisms, the tested rate of degradation may vary with the source of the seawater. Due to the lack of an in‐depth study of degrading strains in the ocean, the selection of microorganisms in artificial seawater is difficult.

## 6 Influence of Degradation Products on the Marine Ecosystem

Degradation of biodegradable polymers may result in total mineralization or partial degradation depending on the molecular structure of the polymer and the present microorganisms (bacteria, fungi, and actinomycetes) capable of using the polymer as a carbon source. Depending on the respiratory conditions (aerobic/anaerobic) and the microorganisms involved, different biogas (CO_2_, NH_3_, CH_4_, H_2_S, or H_2_) or other organic degradation products might be produced.^[^ ^54^, ^136^ ^]^ In general, contrary to many other chemicals, the environmental impact assessment of polymers is not generally covered by laws such as the European legislation on chemicals (REACH). Therefore, ecotoxicological data for biodegradable polymers is scarce. With recent research focused on the biodegradability of polymers in marine environment, the impact of degradation products on the marine ecosystem is unknown. More studies have been carried out in the field of human toxicology, as biodegradable polymers are often used in medical applications. Final production and byproducts of degradation in natural seawater and their impacts on the multispecies communities and biogeochemical processes, such as elemental cycling are not addressed by existing test procedures, or included as part of active biodegradability standards or test methods developed for aquatic ecosystems. In contrast, for compostable plastics, certain standards and norms have been established, which also include ecotoxicity requirements. The European standard EN 13432, for example, requires data on the germination and growth of plants.^[^ ^137^ ^]^

To date, different organisms are used in the lab to assess toxicity of degradation products: for terrestrial ecosystems different plant species and microorganisms are applied. During the degradation process, a generally increased microbial activity (accompanied by a drop in pH value and abnormal high oxygen demand) can have a temporary negative impact on soil organisms, similar studies need to be developed for marine organisms.^[^ ^138^ ^]^ For example, the degradation process of PBAT was monitored in aqueous medium and no adverse effects on luminescent bacteria (light emission) and crustacean Daphnia magna (mobility) was detected.^[^ ^139^ ^]^ In addition, no effects on luminescent bacteria was proven during the degradation of modified starch‐cellulose fiber composites after sieved trough 0.25 mm and 0.75 mm membraned and incubation in aqueous medium (100 mg L^−1^) for 48 d.^[^ ^140^ ^]^

In contrast, for commodity polymers some papers prove an adverse effect on certain organisms: Polyethylene and poly(vinyl chloride), but also poly(lactic acid) polymer microbeads were found to affect the feeding behavior of the lugworm *Arenicola marina*.^[^ ^141^ ^]^ Exposure to polystyrene nanoparticles has been shown to reduce population growth in the algal species *Scenedesmus obliquus*, and to affect the body size and reproductive behavior of *Daphnia magna*.^[^ ^142^ ^]^ Further, exposure to polystyrene microplastics has been found to influence the feeding behavior and reproductive output of the copepod *Calanus helgolandicus*.^[^ ^143^ ^]^ Further studies need to be conducted to assess the impact on degradation products on the marine ecosystem.

## 7 Conclusion and Prospects

The development of seawater‐degradable polymers is challenging but would probably play an important part in the solution to the plastic waste problem. With only slightly basic pH‐value, high salt content, and mostly relatively low temperature, seawater‐degradation either need to rely on quick hydrolysis or on selective enzymatic cleavage of the polymers or other orthogonal stimuli, which need to be developed.

Despite these obvious challenges, polymer chemistry offers the potential to develop polymers, copolymers, and polymer blends with useful properties (such as mechanical stability or barrier properties) that proved reasonable degradation rates in seawater. Promising approaches combine quickly enzymatically degradable polymers, such as starch, PGA, or PVA, that are blended into polyester matrices. After their degradation, the matrix left behind exhibits increased surface area, which proved increased degradation rates compared to the same bulk polymer. Besides blending, also chemical handles can be installed that increase hydrophilicity or decrease hydrolytic stability of the polymer (or copolymer), which eventually results in a degradation in seawater by hydrolysis or in combination with a certain microbial attack. A recent data‐driven approach elucidated degradation trends of plastic debris by linking abiotic and biotic degradation behavior in seawater with physical properties and molecular structures.^[^ ^144^ ^]^ The results reveal a hierarchy of predictors to validate degradation kinetics; we believe this is a pioneering approach that will help the design of novel seawater‐degradable polymers. With very promising properties in the packaging fields, especially P3HB may be an important future material, which also undergoes seawater‐degradation in reasonable times. However, the bacterial polymer is expensive due to complicated extraction processes. Novel chemistries have been very recently developed and we are sure that those will foster the use of P3HB in the polymer industry.

Another challenge is to consider also the degradation products, which should not be toxic. Only few studies, to date, consider toxicity of degradation products for the marine environment, which is still unknown for most polymer fragments and also additives and needs to be tackled with future research. In summary, the development of seawater‐degradable polymers is possible but can only be part of the fight against the worldwide waste problem. Reduce, reuse, and recycle are the major strategies that will help to tackle the increasing plastic waste amounts in the nature environment. However, in some applications a desired and, in the best case, programmed degradation in seawater might be desirable, for example, for packaging or plastic products that have high chances to end up in the ocean. Such materials should exhibit high stability during their use but fast degradation once they enter marine environment to prevent the formation of microplastics. To achieve these goals, research needs to make sure that such safely degrading materials, for example, in packaging, will not be misused and intentionally littered; the reduction of unnecessary plastic products shall still be the first paradigm. Finally, changing people's mind is a never ending story—value the oceans and do not litter.

## Conflict of Interest

The authors declare no conflict of interest.
